# How protective is cervical cancer screening against cervical cancer mortality in developing countries? The Colombian case

**DOI:** 10.1186/1472-6963-10-270

**Published:** 2010-09-16

**Authors:** Luz Angela Chocontá-Piraquive, Nelson Alvis-Guzman, Fernando De la Hoz-Restrepo

**Affiliations:** 1 Public Health Department, Universidad Nacional de Colombia, Bogotá, Colombia; 2 Economic Science Faculty, Universidad de Cartagena, Colombia

## Abstract

**Background:**

Cervical cancer is one of the top causes of cancer morbidity and mortality in Colombia despite the existence of a national preventive program. Screening coverage with cervical cytology does not explain the lack of success of the program in reducing incidence and mortality rates by cervical cancer. To address this problem an ecological analysis, at department level, was carried out in Colombia to assess the relationship between cervical screening characteristics and cervical cancer mortality rates.

**Methods:**

Mortality rates by cervical cancer were estimated at the department level for the period 2000-2005. Levels of mortality rates were compared to cervical screening coverage and other characteristics of the program. A Poisson regression was used to estimate the effect of different dimensions of program performance on mortality by cervical cancer.

**Results:**

Screening coverage ranged from 28.7% to 65.6% by department but increases on this variable were not related to decreases in mortality rates. A significant reduction in mortality was found in departments where a higher proportion of women looked for medical advice when abnormal findings were reported in Pap smears. Geographic areas where a higher proportion of women lack health insurance had higher rates of mortality by cervical cancer.

**Conclusions:**

These results suggest that coverage is not adequate to prevent mortality due to cervical cancer if women with abnormal results are not provided with adequate follow up and treatment. The role of different dimensions of health care such as insurance coverage, quality of care, and barriers for accessing health care needs to be evaluated and addressed in future studies.

## Background

Cervical screening is considered a highly effective intervention that has led to a 70% reduction in mortality by cervical cancer in developed countries [1]. By contrast, Latin America and the Caribbean have failed to impact cervical cancer mortality rates in spite of the implementation of universal screening [2]. Incidence rates of cervical cancer in Latin America and the Caribbean (age adjusted rate = 29.2 per 10^5^) are higher than in any other region in the world except Africa [3].

Colombia has a cervical cancer screening program since 1975 but it has been unsuccessful in significantly reducing cervical cancer incidence or mortality [4]. One of the potential causes for this failure is the introduction of managed health care system in 1993. The Colombian health system is supposed to provide universal health insurance coverage within two regimes: 1) The Contributory Regime, mainly administered by private companies, that covers formally employed and independent workers who contribute to the scheme. Contributions are collected by the insurer of choice. 2) The Subsidized Regime, administered by public institutions or private companies, covers the poor and indigent individuals who cannot afford to make any insurance contribution. Both regimes have a basic benefits package, but the mandatory health care plan (POS, sp. Plan Obligatorio de Salud) for the contributory regime includes every level of care while the mandatory health care plan for the subsidized (POSS, sp. Plan Obligatorio de Salud Subsidiado) is complemented with services provided by public hospitals and financed through traditional supply-side subsidies [5]. In this system, there is considerable fragmentation of activities between private and public actors with a weak role of supervision and regulation by the Ministry of Health. In addition, it provides little room for centralized activities and the cervical cancer screening program has been unable to adapt to this changing reality[6].

There are, at least, two critical components of a cervical screening program, coverage and follow up activities. Several reports have described that ensuring an adequate follow up of women with Pap smears abnormalities is very important to decrease the incidence of cervical cancer and deaths due to it [7]. Yabroff et al (2003) identified barriers of access to follow up after abnormal screening results arising from health providers, patients and from the health system itself[8]. The likelihood of detecting cervical cancer in advanced stages or even dying due to it is increased by those barriers.

Screening coverage is commonly used as the only indicator to evaluate the success of cervical cancer screening programs. However, screening is just a part of them, therefore screening programs must be evaluated for the full range of services needed to prevent cervical cancer and reduce cervical cancer mortality. In the present study, a range of data sources related to quality of the cervical cancer screening program in Colombia were analyzed to provide evidence for our argument.

## Methods

Colombia is a middle income South American country with 46 million inhabitants. It is divided in 32 departments plus the capital district, Bogotá.

### Study design

An ecological analysis was carried out comparing rates of cervical cancer mortality with screening coverage and follow up indicators at the departmental level.

### Dependent variable

Mortality rates by cervical cancer were estimated using the 2000-2004 mortality records from the National Department of Statistics (DANE, sp. DepartamentoAdministrativoNacional de Estadisticas). National mortality information is routinely collected and analyzed by DANE using individual records of every person deceased. DANE makes estimations of under registry by department using information collected from national census [9]. Mortality rates were adjusted using the Bennett- Horiuchi method. The following codes of death were included: C530, Malignant neoplasm of the endocervix; C531, Malignant neoplasm of the exocervix; C538, Overlapping lesion of cervix uteri; C539, Cervix uteri, unspecified; and C55X, Malignant neoplasm of uterus, part unspecified (10th ICD). To address potential misclassification bias the following algorithm was used, cervical cancer deaths = cervical cancer deaths registered+ α* deaths by uterine cancer of unspecified part+ β*deaths by cancer of corpus uteri; α = 0.9 and β = 0.3. Values for *α *and *β *were obtained and adapted from Loos et al [10].

Variables related to cervical cancer screening. The following variables were extracted from the National Survey of Demography and Health (ENDS, sp. Encuesta Nacional de Demografía y Salud) [11], all proportions were estimated by department: proportion of women who reported having a cervical cytology once a year, proportion of women who have never had a cervical cytology, proportion of women with an abnormal cervical cytology who contacted their health care provider to receive treatment and proportion of women who did not collect the results of their last cytology. The ENDS is carried out every five years using a stratified polietapic probabilistic sample of 37,211 households. In 2005, the survey collected information from 41,344 women within ages 13 to 49 years and had a response rate from 88% to 92% depending on the geographical unit. An additional variable included in the analysis was the proportion of women covered by any of the health insurance regimes by department. Health insurance status was used to adjust the effect of screening variables on mortality since access to screening and mortality may vary by insurance status.

### Analysis

The 33 departments were classified according to values of mortality and screening characteristics. They were grouped within 4 categories according to the frequency of cervical cancer mortality (0-9.9 per10^5^, 10.4-12.7 per10^5^, 12.9-15.6 per 10^5^, and 16.0-21.8 per10^5^.) Variables related to cervical cancer screening were also categorized by quartiles. Tables were used to make cross comparative analysis between mortality and cervical cancer screening characteristics. Poisson regression models were built to examine the strength of the association between rates of mortality and categories of screening coverage, proportion of women looking for treatment, and proportion of women who do not collect results. Results of Poisson regression are given as incidence rate ratios (IRR) with 95% confidence intervals[12].

Since we used aggregated national data we were not required to be approved by an institutional review board.

## Results

Between 2000 and 2004, an average of 2918 annual deaths due to cervical cancer was reported by DANE (Table 1). The proportion of women undertaking a conventional cervical cytology once a year was 47.5%. Only 13.7% of the women interviewed have never had a pap smear.

**Table 1:** Distribution of cervical cancer mortality rates (2000-2004) and indicators of cervical cancer screening programs

Department	Women 18 to 69 years old	Average of deaths by year	Mortality per 100,000	Screening variables (%)				
				**1**	**2**	**3**	**4**	**5**

**Amazonas**	14,633	5	16.0	39.4	9.7	87.7	17.2	25.4

**Antioquia**	1,695,093	353	12.7	47.3	6.7	88.4	8.9	28.3

**Arauca**	58,004	16	14.5	50.2	7.3	86.5	18.5	26.1

**Atlántico**	627,571	143	13.5	38.9	6.4	85,7	20.3	35.2

**Bogotá**	2,173,732	473	13.9	54.3	8.9	74.2	10.1	18.9

**Bolívar**	514,680	98	10.7	36.7	4.9	82.9	19.8	38.6

**Boyacá**	357,138	65	10.4	43.1	17.4	64.6	17	21

**Caldas**	298,632	107	21.8	57.3	12.5	82.4	10.9	31.6

**Caquetá**	104,560	27	13.5	44.3	12.0	79.0	17.5	41

**Casanare**	72,240	15	11.1	51.9	7.4	88.6	16.2	37

**Cauca**	336,097	78	12.7	49.9	15.6	83.8	21.1	42.1

**Cesar**	231,167	56	12.9	34.8	7.5	86.6	18.2	38

**Chocó**	104,910	10	4.5	36.1	9.0	84.2	16.4	37.4

**Córdoba**	384,543	87	12.4	34.8	3.9	87.5	16.5	48.7

**Cundinamarca**	618,658	97	9.0	49.7	10.2	81.3	15.8	19.4

**Guainía**	7,514	1	6.3	37.0	14.9	100.0	14.3	14.8

**Guaviare**	19,799	3	7.1	40.7	12.8	88.9	12.5	19.2

**Huila**	263,798	79	16.4	48.2	10.8	73.6	12.8	42.4

**La Guajira**	157,054	19	6.3	28.7	5.0	88.3	18.3	40.2

**Magdalena**	299,679	63	11.3	30.9	9.8	88.1	22.3	45.2

**Meta**	206,687	71	19.7	46.4	10.9	84.1	12.8	36.8

**Nariño**	417,498	105	14.1	54.7	13.9	81.0	15.5	24.2

**Norte de Santander**	345,178	95	15.6	40.9	5.5	83.2	20.3	45.4

**Putumayo**	73,462	10	6.8	65.6	5.1	90.4	10.4	23.7

**Quindío**	165,207	56	20.9	61.9	8.4	82.9	9.6	31

**Risaralda**	278,130	90	20.0	51.4	9.3	83.0	9	37.4

**San Andrés Island**	21,356	3	8.7	59.9	6.9	71.4	9.4	12.8

**Santander**	580,869	140	14.3	52.4	11.1	83.3	12.4	26.8

**Sucre**	201,907	46	12.4	32.1	5.3	78.3	19.8	40.2

**Tolima**	381,515	141	21.1	44.7	7.0	76.5	15.3	38

**Valle**	1,273,844	365	17.6	48.8	6.0	85.5	10.2	30.7

**Vaupés**	8,492	0	0.0	33.1	13.4	100.0	22.4	9.6

**Vichada**	11,862	1	3.9	45.2	11.7	90.2	10.3	22.2

1. Proportion of women who reported having a cervical cytology once a year

2. Proportion of women who did not collect the results of their last cytology

3. Proportion of women with an abnormal cervical cytology who contacted their health care provider to receive treatment

4. Proportion of women who have never had a cervical cytology

5. Proportion of uninsured population

Most women collected the results of cervical screening and looked for medical advice when cervical abnormalities were reported. The largest proportion of women who failed to collect the results was in Boyacá (17.4%) which also had the lowest proportion of women looking for medical advice when cervical abnormalities were reported (64%) (Table 1).

Cervical cancer mortality rates did not decrease in those departments where more women were covered by screening. By contrast, the proportion of women looking for treatment after an abnormal result was associated moderately with mortality by cervical cancer. A protective effect was observed when the proportion of women looking for treatment was higher than 81%, IRR 0.65 IC95 % (0.56 - 0.76), this association remained significant after adjusting by screening coverage or health insurance coverage. An important increase in cervical cancer mortality was observed in areas where the proportion of women without health insurance was higher than 22%, IRR 1.66 IC95% (1.42 - 1.95);(Table 2).

**Table 2:** Results of Poisson regression analysis given as incidence rate ratios (IRR)

Range	Mortality Rate by cervical cancer	IRR (CI 95%)	Adjusted IRR (CI 95%) *
**1. Proportion of women who have never had a cervical cytology**			
**8.9 - 10.4**	22.5	-	

**10.9 - 15.5**	19.9	1.05 (0.95 - 1.15)	0.86 (0.78 - 0.96)

**15.8 - 18.2**	25.5	0.82 (0.72 - 0.93)	0.68(0.57 - 0.80)

**18.3 - 22.4**	24.3	0.93 (0.84 - 1.02)	0.71(0.61 - 0.82)

**2. Proportion of women who had an abnormal cervical cytology and contacted their health care provider to receive treatment**			
**64.6 - 81.0**	23.9	-	-

**81.3 - 83.8**	24.3	1.01 (0.92 - 1.11)	0.88(0.79 - 0.98)

**84.1 - 87.7**	25.8	1.08 (0.98 - 1.18)	0.88 (0.77 - 1.01)

**88.1 - 100**	19.9	0.83 (0.74 - 0.93)	0.65 (0.56 - 0.76)

**3. Proportion of uninsured women**			
**9.6 - 22.2**	20.0	-	-

**23.7 - 31.0**	21.2	1.23 (1.11 - 1.35)	1.47 (1.27 - 1.69)

**31.6 - 38.0**	22.3	1.44 (1.29 - 1.61)	1.77 (1.54 - 2.03)

**38.6 - 48.7**	23.4	1.14 (1.02 - 1.27)	1.66 (1.42 - 1.95)

**4. Proportion of women who have a cervical cytology once a year**			
**28.7 - 36.7**	19.9	-	
	
**37.0 - 44.7**	25.8	1.30 (1,13 - 1,48)	
	
**45.2 - 50.2**	23.7	1.19 (1,06 - 1,34)	
	
**51.4 - 65.6**	24.5	1.23 (1,09 - 1,38)	
	
**5. Proportion of women who did not collect the results of their last cytology**			
**3.9 - 6.4**	24.0	-	
	
**6.7 - 8.9**	23.2	0.96 (0,88 - 1,05)	
	
**9.0 - 11.1**	23.4	0.98 (0,88 - 1,08)	
	
**11.7 - 17.4**	25.2	1.04 (0,92 - 1,19)	

*Adjusted by variables 1, 2, 3 included in the final model.

## Discussion and Conclusions

This analysis suggests that cervical cancer screening coverage was not protective against cervical cancer mortality in Colombia as it has been shown in several reports [4,13,14]. Instead, the proportion of women who complied with follow up after an abnormal cytology was statistically associated to cancer mortality. After adjusting by health insurance coverage, those areas with the highest percent of women who contacted their health care provider after an abnormal result had almost 40% less mortality than the areas with the lowest proportion.

Several alternatives have been proposed to increase the adherence to follow up and treatment of women with an abnormal result in a Pap smear. Yabroff et al (2000) conducted a meta-analysis of different strategies to increase compliance to follow up after an abnormal cervical cancer screening. They found that cognitive interventions, telephone confirmation of appointments, were more effective than sociological or behavioral interventions. Combining behavioral, cognitive and sociological strategies yielded inconsistent results. Individual studies also support the efficacy of cognitive interventions [15]. Engelstead et al tested a combination of outreach community visits with counseling and telephone calls in a group of women in Oakland CA, USA. They found that this strategy increased the adherence to follow up and abnormalities treatment by more than 90%[7]. Marcus et al reported that providing women with transportation incentives and a personalized follow up letter also increase adherence to follow up visits[16].

One potential weakness in this study is the quality of vital statistics since misclassification in death causes may occur. However, more than 90% of deaths by uterine cancer in Colombia were classified as cervical cancer which made that explanation implausible. On the other hand, women answering the survey may have given equivocal answers to questions about use of health service related to cervical cancer screening, this potential source of error, would produce a non-differential misclassification bias resulting in an underestimation of the association between cytology coverage and cervical cancer mortality. Nonetheless, ENDS surveys have been carried out for more than 20 years in Colombia using similar instruments; surveyors are well trained and have experience applying these questions. In addition, Caplan et al 2003 have found that self-reporting of cervical cancer screening test utilization is highly accurate compared with information extracted from medical records (overall agreement > 80%) [17]. Therefore, we believe that self-reporting bias on use of screening services would be unlikely to mask a strong effect of cytology coverage on cervical cancer mortality in our study.

A potential explanation for the lack of relationship between coverage and cervical cancer mortality lies on how the Colombian Health system is structured. People in the contributory regime receive the most complete package of services while those in the subsidized regime are entitled to receive a more limited package of services. Until 2005 colposcopy and cervix biopsy were not provided to subsidized or uninsured people. Yabroff et al (2003) has reported that there are barriers for access to follow up after cervical screening that arise from health providers. Two of the most important are being uninsured or underinsured, and the perception that follow up procedures are too expensive. Specific studies on barriers for access to follow up after cervical screening are needed in Latin America [8]

Recent studies have addressed the deleterious effect of the health care reform in Colombia upon several indicators of health situation. Ruiz et al has described how the Colombian health system has been unable to reduce maternal mortality while Chile, where public hospitals have been strengthened, has lowered mortality rates by more than 70% [18]. However, we were unable to formally test the role of health providers in the magnitude of death rate by cervical cancer since we had no access to information on the health insurance status of individual women. This is another potential shortcoming of our analysis.

The results presented here suggest that cervical cancer screening programs should not be evaluated based on the number of smears taken per year only. Other indicators such as quality of smear samples, proportion of cervical lesions confirmed using biopsy or colposcopy, compliance with follow up, and percent of women effectively treated among those with cervical abnormalities should also be used. Some have proposed HPV detection as a complementary service to improve cervical cancer screening. Gamboa et al [19]. assessed the cost effectiveness of introducing HPV DNA detection tests in Colombia concluding that it would reduce cervical cancer mortality and would be very cost-effective. However, including the test would only complicate further the screening process that already seems to be failing in providing basic services of follow up.

Colombia is currently considering the introduction of HPV vaccination in girls as a measure to improve the prevention and control of cervical cancer. One important barrier for HPV vaccination in middle income countries is the current price of available HPV vaccines. Goldie et al (2007) has shown that for an average Latin American country prices above USD$50 per vaccine dose are hard to be affordable though vaccination would be cost effective [20]. Other aspect to consider is whether a booster of vaccine is needed to meet the goal of cervical cancer reduction. If vaccine boosters are needed then the cost effectiveness ratio of vaccines would become less attractive. Once vaccine prices become more affordable, a combination of universal vaccination and strengthening of the cervical cancer screening program would become the ideal health policy to control cervical cancer.

## Competing interests

Fernando de la Hoz has been consultant for Glaxo Smith Kline Division of Vaccines in Colombia. Luz Angela Chocontá and Nelson Alvis have no conflict of interest.

## Authors' contributions

LAC participated in the design of the study, collected the information, performed the statistical analysis and wrote a draft of the manuscript. FH conceived and designed the study, gave advice on the analysis and participated in writing the final manuscript. NA gave advice on the design of the study, assessed the quality of the information on mortality and helped to write the manuscript. All authors read and approved the final manuscript

## Pre-publication history

The pre-publication history for this paper can be accessed here:

http://www.biomedcentral.com/1472-6963/10/270/prepub
